# Perceptions of Barriers to Using Opioid Analgesics: A Mixed Methods Study

**DOI:** 10.1089/pmr.2023.0021

**Published:** 2023-08-30

**Authors:** Atsede Aregay, Margaret O'Connor, Jill Stow, Nicola Ayers, Susan Lee

**Affiliations:** ^1^ Department of Health and Nursing Sciences, University of Agder, Kristiansand, Norway.; ^2^ School of Nursing, Mekelle University, Tigray, Ethiopia.; ^3^ School of Nursing and Midwifery, Monash University, Melbourne, Victoria, Australia.; ^4^ Department of Palliative Care, Melbourne City Mission Palliative Care, Melbourne, Victoria, Australia.; ^5^ Department of Perioperative Medicine, St Vincent's Private Hospital, Melbourne, Victoria, Australia.; ^6^ School of Nursing, BPP University, London, United Kingdom.; ^7^ School of Nursing and Midwifery, Monash University, Melbourne, Victoria, Australia.

**Keywords:** accessibility, availability, mixed-method study, morphine, opioids, rural

## Abstract

**Background:**

Availability and accessibility of opioids are a worldwide problem. In low-resource settings, such as Ethiopia, access to opioids is either limited or nonexistent and legally restricted in health care settings. This study aimed to identify barriers for the availability and accessibility of opioids in Ethiopian rural and regional health care settings.

**Methods:**

A mixed-method case study design was used. A total of 220 nurses from primary, secondary, and tertiary health care settings were invited to participate in a survey of knowledge and practice. For the qualitative interview, 38 participants were recruited from educational facilities, health services, and the community across a region.

**Results:**

Barriers in availability and accessibility of opioid analgesics were expressing pain considered as a sign of weakness, lack of knowledge, side effect concerns about prescribing morphine, only doctors being authorized to prescribe morphine, lack of foreign currency to import morphine ingredients, and inequity in accessing morphine in hospitals and none in rural health care settings.

**Conclusion:**

The findings of this study indicate that opioids, particularly morphine, were not consistently available and accessible to all patients in need. Health professionals lacked knowledge about opioids. Strengthening the existing pain-free initiatives and improving the type, dose, and supply of morphine could help reduce needless suffering and enhance access to essential pain medicines for those in need.

## Introduction

According to the World Health Organization (WHO), it is estimated that each year 6.5 million people suffer with moderate-to-severe pain.^1^ Relief of this pain cannot be achieved without improving the availability and accessibility of opioid analgesics.^2,3^ Strong opioid analgesics including morphine are effective for the treatment of moderate-to-severe pain.^3^ Despite morphine being inexpensive and included in the WHO model list of essential medicines, its availability is a major international problem, particularly in low- and middle-income countries (LMICs).^3–6^

Globally, >5 billion people do not have access to essential pain relief medications including opioids.^7,8^ More than 90% of the world's opioids are consumed in high-income countries, accounting for <20% of the world's population.^7^ In LMICs, opioids are either nonexistent, legally restricted, or accessed by a fraction of the population.^5,7^ For example, in Uganda, where oral morphine is manufactured locally, it is only accessible for 2.3% of the population in need.^8^

Several barriers to access opioid analgesics were identified in a number of African countries,^8,9^ including overly restrictive medicine laws, insufficient knowledge of health care providers, lack of budget to import opioid, prescribers' fear of making mistakes, and restrictive prescription regulations (only doctors being permitted to prescribe).^9^ In Ethiopia, although morphine is included in the list of essential medicines, millions of people with chronic diseases do not have access to opioids.^5,10–12^ Therefore, this study aimed to identify barriers for the availability and accessibility of opioids in rural and regional health care settings.

## Methods

### Study design and population

**FIG. 1.**
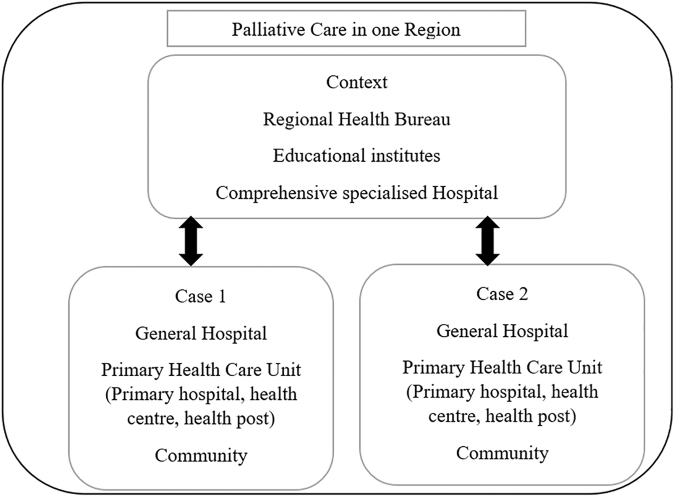
A case study design and embedded units of the Context, Case 1, and Case 2.

The study was undertaken in 1 of the 11 Ethiopian states in 2018. A multiple-embedded mixed-method case study design was applied^13^ and the case represented palliative care in one region of Ethiopia. The Context and the two cases (Case 1 and Case 2) with embedded subunits were two zones of the region (Fig. 1). A total of 220 nurses were invited to participate in a survey that included a knowledge test as part of the mixed-method study. Face-to-face in-depth interviews were also conducted with 38 participants, including nurses (Table 1), and this article reports on the analysis of these interviews and the survey data.

**Table 1.** Number of Interview Participants from the Context, Case 1, and Case 2

Working area	Participants
Context: 12 interviews	Two regional health bureau Four school heads (medicine, nursing, and pharmacy) at university, college of health sciences, and regional health science college Three head nurses (medical, surgical, and oncology) in comprehensive specialized hospital Three (one head pharmacy, one CNO, one MD) in comprehensive specialized hospital
Case 1: 12 interviews	Five (one head pharmacy, one CNO, one MD, two head nurses) in general hospital Four (one head pharmacy, one CNO, one MD, one head nurse) in primary hospital Two (one head pharmacy, one MD) in health center, one health extension worker
Case 2: 14 interviews	Seven (one head pharmacy, one CNO, one MD, two head nurses, two head doctors) in general hospital Four (one head pharmacy, one CNO, one MD, one head nurses) in primary hospital Two (one head pharmacy, one MD) in health center one health extension worker
Case 1 and Case 2	Two community groups in rural areas

CNO, chief nursing officer; MD, medical director.

### Data collection and analysis

The survey was adapted from the Palliative Care Quiz for Nursing (PCQN) for the knowledge test,^14^ the quantitative results are reported fully elsewhere.^15^ Seven pain and symptom management items were used from PCQN. The interview guide was developed from the WHO public health strategy.^2^ Focus group interviews were conducted among rural community members to share their experience of pain and its management. To analyze the survey data, descriptive statistics were computed, with interviews transcribed verbatim, translated, and analyzed using NVivo 12. A thematic analysis technique was used.^16^

All methods of the study were performed in accordance with the Declaration of Helsinki.^17^ Interview participants provided written informed consent. The return of the survey was considered implied consent. Interview recordings were deidentified to maintain confidentiality. The study was approved by an Ethiopian Ethical Review Committee (1494) and Monash University (13402), Australia.

## Results

### Accessibility and availability status of opioid medicines

Availability and administration of opioids and nurses' self-reported practice in administering pain medication, including morphine, are described as follows.

A representative from the regional health bureau indicated that morphine is only available in hospitals: “*…* [morphine] is *available in the … primary, general hospitals and comprehensive specialised Hospital.*” (Participant CRHBR). A health center director added that “*… we have a standard issued by the regional health bureau on what medications should be available in health centres, primary, and general hospital. So, we do not keep morphine* [in the health centres and health posts] *…*” (Participant 2HCHO).

Nurses are responsible for administering prescribed medications; however, they noted that although they administered prescribed drugs including morphine without formal training: “*…nurses are not trained to administer it* [morphine] *…*” (Participant CTHWNH3). Teamwork and on-the-job training were instrumental in bridging the gaps: “*… having proper* [training] on *administering morphine is necessary, but we have a clinical pharmacist with us… We work in a team … and administer the medicine with guidance…*” (Participant CTHWNH2).

The quantitative survey among nurses showed that 99 (57%) out of 173 nurses reported conducting pain assessments. In the Context group, 52 (58%) and 24 (49%) from Case 2 reported administering “morphine” in the past year. For Case 1, the most common medication administered was “tramadol” 89% (Table 2).

**Table 2.** Self-Reported Practice of Nurses Toward Palliative Care (*n* = 173)

Items	Context (%)	Case 1 (%)	Case 2 (%)
	Yes	Yes	Yes
Pain assessment and management			
Pain assessment			
Assessed pain at least once	50.6	62.9	65.3
Administered pain medication at least once			
Administered morphine	58.4	5.7	49.0
Administered tramadol	62.9	88.6	75.5
Administered paracetamol	41.6	54.3	46.9
Applied nonpharmacological intervention at least once			
Applied hot or cold compress	51.7	71.4	59.2
Applied massage	32.6	20.0	59.2
Used distraction	57.3	65.7	67.3
Total sample (*n*)	89	35	49

### Cultural attitudes toward pain and pain management

Culturally, people are expected not to complain about their pain. Health professionals often invoked historical and cultural contexts to discourage patients who wanted to discuss their pain. “*If a patient complained about their pain …, the physicians … told the patient,* ‘*Are you not an adult’… you know your ancestors passed through lots of hardships and war; why do you complain about this simple pain …*” (Participant CTHP). Participants indicated cultural values and community narratives about pain. “*… if someone is suffering with pain, they* [people in the community] *say to them ‘strong’ people tolerate pain*” (Participant CTHP).

An NGO representative added “*… pain is ignored in our country … they say are you not a ‘man’, meaning aren't you strong*. *Also, if you ask an older adult* [senior citizen] *… they* [consider it as] *part of the aging process … The community's … think pain is part of life and discourage those who talk about their pain*.” (Participant CNGOR).

However, some participants from Case 1 reported that nurses practice: “*… pain management…*[which] *is considered the fifth vital sign. So, we conduct pain assessment and manage the pain of the chronic patients …*” (Participant 1GHND).

### Knowledge gaps in pain management

Ethiopia has a Pain-Free Hospital Initiative implemented in different hospitals, including in the comprehensive specialized hospital of the study setting. “*… there is a country-wide initiative … called pain-free hospitals, led by Ministry of Health … For example, the program has been successfully implemented by Menelik Hospital. We learned from their experience and applied it to our institution.*” (Participant CTHD), a staff of the comprehensive specialized hospital, said.

Participants from the Context, Case 1, and Case 2 stated that the pain management guidelines were followed: “*… we usually follow WHO's pain management guideline … and our system allows for the provision of pain medications up to a level of morphine…* (Participant 2GHD1). However, as the survey indicates, five out of the seven PCQN pain management questions were scored correctly by 50% or less of the surveyed nurses. Question 3 (Table 3) had the lowest score in the Context, Case 1, and Case 2, respectively (6%, 3%, and 10%).

**Table 3.** Items from the Palliative Care Quiz for Nursing With the Highest and Lowest Percentages of Correct Responses *(n [Context = 87, Case 1 = 35, and Case 2 = 49])*

No.	Items	Correct responses ***n*** (%)	Correct responses ***n*** (%)	Correct responses ***n*** (%)
		Context	Case 1	Case 2
Pain management				
Q 3	The extent of the disease determines the method of pain treatment (F).	5 (5.7)	1 (2.9)	5 (10.2)
Q 4	Adjuvant therapies (antidepressant, anticonvulsant, and antiemetics) are essential in managing pain (T).	47 (54.0)	16 (45.7)	17 (34.7)
Q 7	Drug addiction is the major problem when morphine is used on a long-term basis for the management of pain (F).	11 (12.6)	5 (14.3)	10 (20.4)
Q10	During the terminal stages of an illness, drugs that can cause respiratory depression are appropriate for the treatment of severe dyspnea (T).	57 (65.5)	22 (62.9)	32 (65.3)
Q 13	The use of placebos is appropriate in the treatment of some types of pain (F).	37 (42.5)	14 (40.0)	23 (46.9)
Q 16	Meperidine (Demerol) is not an effective analgesic in the control of chronic pain (T).	18 (20.7)	11 (31.4)	12 (24.5)
Q 18	The manifestation of chronic pain is different from those of acute pain (T).	74 (85.1)	24 (68.6)	42 (85.7)

In total, 51 (58.6) in the Context, 20 (57.1) in Case 1, and 24 (49.0) in Case 2 do not know Q 16, and T: True and F: False.

In the comprehensive specialized hospital and regional health bureau staff acknowledged that “*…we have trained at least one person from every department or profession … nurses, pharmacy and even physicians….*” [However]*, we have not trained everyone yet.* (Participant CTHP) (Table 3).

### Concerns and reservations about prescribing opioids

Views on the side effects of morphine and other opioids were divided. A pharmacist from Case 1 facility expressed that “*… when patients have severe pain, the doctors in our facility* [a general hospital] *prescribe pethidine because morphine is considered unsafe due to its side effects*.” (Participant 1GHP). However, the medical leader from the same institution refuted the statement. “*… we do not prescribe pethidine for pain management. Pethidine is not recommended as it is addictive… morphine is the only safe drug*” (Participant 1GHD).

In contradiction, other participants justified using opioids, while acknowledging potential side effects, a doctor from the Context noting, “*… morphine does not have addiction the one that addictive is pethidine. If a person is about to die within three or four months, … I think it is justifiable … to prescribe these drugs whether they cause addiction or not…*” (Participant CSH2).

Participants noted that fear of side effects and a general lack of information about morphine often resulted in medications expiring. “*… some doctors did not know it* [morphine] was available in the hospital*… And even those who knew of its availability were afraid of prescribing it…*” (Participant CTHD). A clinical nurse in Case 2 clarified that “*The doctors usually prescribe mild pain drugs first and then progress to other medications for severe cases… But morphine is a rarely prescribed medication …*” (Participant 2GHWNH2).

Participants justified why doctors fear prescribing morphine: “*Doctors believed that … morphine has a side effect on respiratory distress ….*” (Participant CTHP). The reluctance was more among general practitioners, resulting in morphine appearing to be prescribed only by general hospital specialists. “*… Most of the time, morphine is prescribed by the internist, surgeon, orthopaedics … for inpatients who have severe pain …* [because] *the general practitioners fear the side effects to prescribe morphine …*” (Participant 2GHD1).

However, after receiving pain management training, doctors becoming more confident in prescribing. “*… pain management training … has clarified the previous concerns we had on the side effects of morphine ….*” (Participant CTHD). Yet, some participants questioned the sustainability of the training, which was externally supported, for example, by the Centres for Disease Control (Participant CSH1).

### Affordability and supply

The government-owned pharmaceutical fund and supply agency (PFSA) is responsible for procuring and distributing medications to all health care settings in the country. However, participants from the Context, Case 1, and Case 2 explained that drugs were not consistently available. For example: “*… as a matter of policy, we need to buy medications quarterly … But we purchase medications daily… because when you request a quarter worth of supply of medication from PFSA*, [what] *they give us that lasts for four days or four weeks …*” (Participant CTHP).

A Case 1 pharmacist justified their preference to: “*…buy medication from PFSA every three months,* [because] *their price is low …*” (Participant 1GHP). Another pharmacist from the Context setting argued that the supply issue is because of foreign currency shortages: “*…we have a domestic factory* [morphine manufacturing plant]*. However, … production is slowed due to a shortage of ingredients linked to hard currency shortage as the ingredients are imported.*” (Participant CTHP).

Although comprehensive specialized hospital and Case 2 general hospital pharmacists described the availability of essential medicines, those from Case 1 of the general hospital said that morphine is unavailable. “*… morphine is on the national essential medicine list … but the medicine is not available in our* [general] *hospital or most other general hospitals… nor listed on our … essential medicines list …*” (Participant 1GHD). Others from Case 2 and Context concurred, “*… we do not have a continuous supply of morphine in the hospital. However, certain private pharmacies in the cities stock morphine …*” (Participant CSH2).

Despite supply constraints, participants described morphine as one of the cheapest drugs in the region. “*… morphine is cheap… they* [the community] *can afford it …*” (Participant 1GHD). Others, however, disagreed: “*… the price of* [morphine] *is high compared to people's income…*” (Participant CSH2). However, affordability might be improved by access through health insurance. “*… I don't think affordability is an issue with* [morphine], e*specially if the patients are enrolled in a health insurance …*” (Participant CRHBR).

Participants also complained about the absence of dose options and the difficulty in manually splitting morphine tablets into the prescribed milligrams. For example: “*… The morphine tablet … is* available only in 30 mg doses*…if they have been prescribed 5mg, the patients need to divide* [the tablet] themselves*… the patients may split it into ten or four or any size, which may lead to an under or overdose…*” (Participant CTHP). A primary hospital pharmacist from Case 2 also reported the absence of other forms of morphine. “*… we have morphine in the form of tablets and IV. We do not have a syrup …*” (Participant 2PHHO).

### Alternative pain medications

In the Context, when morphine was unavailable, patients received alternative pain medications, such as “*…tramadol because it is … more available than morphine … We prioritise morphine for our terminally ill patients with severe pain …*” (Participant CTHWNH1). However, in the general and primary hospital in Case 1, the doctor prescribed tramadol because that was the strongest available analgesic: “*… we prescribe tramadol as that is what we have … if the patient pain does not respond with tramadol, they will suffer with pain …*” (Participant 1GHD).

Participants from the Context noted that rural communities have limited access to morphine because staff in “*… health centres or health posts are not allowed to prescribe morphine; only doctors in primary hospitals or higher settings, or GPs* [general practitioners] *can prescribe morphine and other opioids…*” (participant CRHBR). In addition, a Case 2 general hospital participant said, “*… as per the country's drug administration policy, nurses cannot prescribe opioids* [such as pethidine, codeine, morphine]*. But they can prescribe NSAIDs* [such as paracetamol, diclofenac, tramadol]” (Participant 2GHD1).

In contrast, health centers and health posts, which are accessed by most of the country's rural population, participants from Case 1 and Case 2 justified that the primary medication was “*… tramadol, our top anti-pain medication … The health centre standard did not allow us to prescribe it* [morphine] *…if the patient has severe pain, we refer them to the nearby hospitals.*” (Participant 1HCHO).

Health extension workers from both cases reported that the only pain medication available in their health post was paracetamol. “*… We do have patients diagnosed with hypertension … cervical cancer … hepatitis* [with severe pain] *… they took anti-pain* [paracetamol] *medicine from our clinic …*[because] *we only have paracetamol*” (Participant 1HEWHP). Patients in the focus group confirmed that “*… when I feel suffering, I got anti-pain from the health centre, but it is not working… I took the medication though not working for me …*” (Participant 2RCFG).

## Discussion

In Ethiopia, despite opioids being incorporated in the list of essential medicines,^4,10,12^ qualitative findings revealed inconsistent or negligible availability, influencing doctors' ability to prescribe. Access is particularly challenging in rural communities.

The survey results indicate that nurses working directly with patients in primary, secondary, and tertiary health care settings had low levels of knowledge toward pain management, with <50% of surveyed nurses answering correctly to the knowledge test questions. This study reported lower knowledge scores compared with nurses' knowledge in a recent Ethiopian study^18^ and Ugandan study^19^ where 67% of nurses in both studies had good knowledge. The difference between these findings may be due to differences in the study settings and the variety of tools used to assess the knowledge test.

However, the findings of this study are consistent with the studies conducted in Iran^20^ and Baghdad,^21^ where <50% of nurses responded correctly to the knowledge questions. This finding implies that capacity-building activities are required to enhance nurses' knowledge about pain management.

Rural areas and isolated communities are highly disadvantaged because health facilities in those settings are staffed by health care providers (nurses and clinical officers) not permitted to prescribe morphine. This contrasts with experience elsewhere, such as in Uganda and Tanzania, where the community's access to these medications had been ensured by allowing trained nurses to prescribe morphine.^5,8,22^

In Ethiopia, only doctors were authorized to prescribe morphine, consistent with Mozambique and Swaziland.^9^ Participants in Case 1 described tramadol as the strongest pain medication prescribed, supported by the self-reported practice part of the survey, where nurses of Case 1 scored tramadol as the most common pain medication, administered by 89% nurse respondents. The use of tramadol as an alternative pain medication in the absence of morphine is consistent with the African study reported by Yorke et al.^23^ A systematic review of studies conducted in Asia also reported that tramadol was the only strong analgesic available for treating pain.^24^

Participants from Context and Case 2 argued that physicians still feared prescribing morphine because of the side effect of respiratory depressions. This was supported by the survey results, wherein nurses were concerned about drug addiction from using morphine. Similarly, a study conducted in Latin America indicated that one of the main barriers to opioid prescription was fear of the adverse effects on patients.^25^

Similarly, physicians working in several African countries exhibited negative attitudes and fear of prescribing opioid,^8,9,26–28^ indicating that more evidence-based training might be needed to improve the prescribers' attitudes. The qualitative findings of the Context reflected these significant obstacles. The shortage of currency for importing ingredients was also found in Mozambique where morphine importation was compromised when foreign currency was scarce.^9^ Participants from the Context and Case 2 also described the scarcity of alternative doses and forms of morphine, especially morphine syrup. This contrasts with experiences of other African countries, where oral morphine solutions are locally reconstituted and distributed in various concentrations to those in need.^4,8,22^

This study has some limitations. The name of medicine “meperidine (Demerol)” used in the survey PCQN was unfamiliar in Ethiopia as it is known in the country as pethidine. In addition, the findings may not be generalizable to other regions in Ethiopia because the study settings were confined to one region. In addition, the recent war in Ethiopia may have altered the focus and priorities of the national health department, affecting available health services. Despite this, at the time of data collection, the status of pain management in the region was likely to be similar in other areas of the country.

## Conclusion

This study has highlighted that opioids, particularly morphine, were inadequately available and inaccessible to those in need. Barriers identified included lack of knowledge, restrictive prescription regulations, absence of morphine in rural areas, lack of foreign currency to import morphine, and prescribers reluctant to prescribe morphine fearing side effects.

In response to these barriers, there is a need for policy changes, including revisions to the rules and regulation of prescribers and prescribing restrictions, to enable nurses and clinical officers to prescribe opioids across all health care settings. Using opioid distribution modeling demonstrated in neighboring countries, there is opportunity to expand the scope for accessing opioids, and to locally produce morphine. And the knowledge gaps identified in this study need to be in addressed in undergraduate and continuing education in relevant health courses. These initiatives and improving the type, dose, and supply of morphine could enhance access to opioids and help reduce needless suffering for those in need.

## Abbreviations Used

CNO: chief nursing officer
LMICs: low- and middle-income countries
MD: medical director
PCQN: Palliative Care Quiz for Nursing
WHO: World Health Organization
